# Competition for Light Interception in Different Plant Canopy Characteristics of Diverse Cotton Cultivars

**DOI:** 10.3390/genes14020364

**Published:** 2023-01-30

**Authors:** Fahmida Sultana, Washu Dev, Minghua Xin, Yingchun Han, Lu Feng, Yaping Lei, Beifang Yang, Guoping Wang, Xiaofei Li, Zhanbiao Wang, Fangfang Xing, Shiwu Xiong, Yabing Li

**Affiliations:** 1 State Key Laboratory of Cotton Biology, Institute of Cotton Research of Chinese Academy of Agricultural Sciences, Anyang 455000, China; 2 Zhengzhou Research Base, State Key Laboratory of Cotton Biology, Zhengzhou University, Zhengzhou 450001, China

**Keywords:** cotton cultivars, cotton canopy structures, light interception, yield

## Abstract

Identifying the ideal plant nature and canopy structure is of great importance for improving photosynthetic production and the potential action of plants. To address this challenge, an investigation was accomplished in 2018 and 2019 at the Institute of Cotton Research (ICR) of the Chinese Academy of Agricultural Science (CAAS), Henan Province, China. Six cotton varieties with diverse maturities and plant canopy structures were used to evaluate the light interception (LI) in cotton, the leaf area index (LAI), the biomass, and the yield throughout the two years of study. The light spatial distribution in the plant canopy was evaluated using a geographic statistical method, following the increasing quantity of radiation intercepted, which was determined using the rules of Simpson. Compared to the cotton plants with a compact structure, varieties with both a loose and tower design captured a comparatively higher amount of light (average 31.3%) and achieved a higher LAI (average 32.4%), eventually achieving a high yield (average 10.1%). Furthermore, the polynomial correlation revealed a positive relationship between the biomass accumulation in the reproductive parts and canopy-accrued light interception (LI), signifying that light interception is critical for the yield development of cotton. Furthermore, when the leaf area index (LAI) was peaked, radiation interception and biomass reached the highest during the boll-forming stage. These findings will provide guidance on the light distribution in cotton cultivars with an ideal plant structure for light capture development, providing an important foundation for researchers to better manage light and canopies.

## 1. Introduction

Cotton is a major cash crop that is grown all over the world as a fiber source [1]. As the world’s population is increasing, the market value for cotton is also raising, which is becoming increasingly important [2,3]. China accounts for 30% of the total global cotton production [4,5]. Furthermore, Henan is the leading cotton producing provinces, with higher than 400 thousand ha under cultivation [6]. Cotton has an erratic growth pattern, and light plays a crucial role in determining its growth and development. To evaluate the features of light interference, that can extend the potential photosynthetic production of leaves, it is critical to govern the effects of the canopy structure on radiation. The canopy architecture influences the light interception (LI) and the distribution, which is an important factor in modifying the aboveground development and regulating the crop yield [6,7]. Frequent experiments have confirmed that a cotton canopy has a significant impact on radiation interception by changing the distribution of the radiation within a canopy [8,9,10]. To investigate the fundamental processes of light capture and vigor conversion to form biomass, it is critical to recognize the precise and specific features of light dissemination in the cotton canopy. The plant structure is strongly related to its yield and stress tolerance; additionally, the canopy size is an important feature of the cotton canopy [11]. Manually assessing the canopy structure is difficult and inaccurate, but it is also difficult to assess the multidimensional traits, for example the leaf area and volume of the canopy. Field experiments with imaging phenotyping systems can quickly obtain data in the field, allowing for the enumeration and observation of the canopy development.

The crop canopy can be optimized according to requirements to improve both the photosynthetic production and yield [12]. The spatial arrangement of a crop’s canopy is often dependent on the variety of the crop. It is the spatial distribution and collection of all the organs’ morphological characteristics [12,13]. The plant type is critical for solar energy interception and its utilization, which ultimately improves the crops photosynthetic productivity [13]. The plant type alters the canopy structure, which shapes the distribution of the light in the cotton canopy; the light interception aids in increasing the light-energy absorption, which increases the crop yield. Light interception, which serves as an assessment index for the distribution of light in the canopy, determines the plant canopy’s ability to intercept radiation [14]. The ability of the intercepted light is inextricably linked with the canopy arrangement, and an appropriate canopy arrangement is critical to improve the light dissemination in the canopy. The pattern of light distribution is determined by the canopy’s structure, which is columnar, reveals an exposed structure, and develops into the inter space, allowing more radiation to be captured in the lower canopy.

Green plants use the dynamic portion of light or radiation (400–700 nm) for photosynthesis. The ability of the crop canopy to capture Photosynthetically Active Radiation (PAR) influences both the reproductive and vegetative productivity, which is the foundation of cotton and other plant economic yields. According to Louarn G., reproductive and vegetative production are dependent on two factors: the amount of radiation intercepted, and the rate of its conversion of radiation to biomass [15]. A linear relationship exists between the accumulative photosynthetic radiation and the cumulative biomass in the majority of crops. The radiation use efficiency (RUE) [16] is the relationship between the intercepted radiation and the cumulative biomass.

Several crop models were introduced as a result of the rapid development of computer technology that can precisely identify the relationships between various crop growth characteristics. Three-dimensional crop growth models, which have recently been developed, are an efficient device for determining the relation between crops and the surrounding in which they grow, specifically with light interception [17]. Herve Rey demonstrated a method for creating a 3D flower patterns using a tool named AMAPsim, which can calculate the MMP radiation and make calculations to simulate light perception during various plant growth stages and organs [18]. Using 3D image technology, H. Sinoquet presented a method for creating 3D crop patterns that can characterize the distribution of light in plant canopies [19]. Ma also used this model to develop a model for rapidly estimating the light distribution in different layers of cotton canopies using a computer-generated camera [10]. Because of the irregular and discontinuous nature of canopies, measuring the light distribution is difficult. A geographic arithmetic sampling method, following the statistics of spatial distribution, is used to investigate both the radiation changes and arrangement in assorted canopies of cotton varieties with different plant structures [20]. Only limited studies compared the spatial light interception, and the vegetative and reproductive production of plants with various architectures and maturities. 

The objectives of this study were to classify and sort out an ideal cotton cultivar with a better accuracy for capturing solar radiation, and an improved comprehension of the nature of light interception and dry matter accumulation in various cotton cultivars. Specifically, the following:

(1) Evaluate the developing features of different canopy structures throughout the growth period;
(2) Determine the cumulative changes in leaf area index, associated with radiation interception in various cotton cultivars and biomass.

## 2. Materials and Methods

### 2.1. Experimental Site

**Figure 1:**
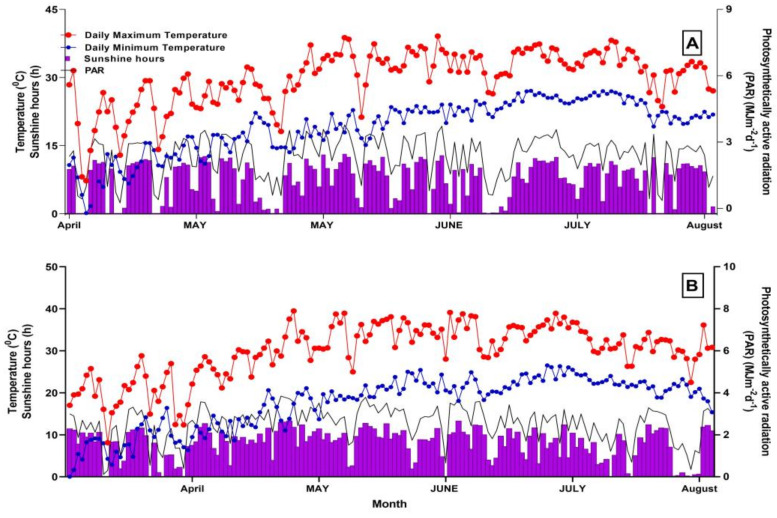
Monthly meteorological conditions during cotton growing season in 2018 and 2019 (**A**, **B**).

This research was carried out in 2018–2019 at the ICR field in Anyang, Henan, China. The experimental field is located at (36°06′ N, 114°21′ E). The land was a medium loam with 0.65 g kg^−1^ N, 0.01 g kg^−1^ P, and 0.11 g kg^−1^ K. During the growing season of cotton (April to October), the average temperature was 24 °C in 2018 and 22 °C in 2019. At the same time, the yearly rainfall was 602 (mm) in 2018 and 579 (mm) in 2019. The date of sowing in 2018 was 20th April and in 2019 was 18th April. Every year, the same field was used, and the soil was medium loam. The daily minimum and maximum temperatures, sunshine hours, and Photosynthetically Active Radiation (PAR) are presented in Figure 1, which are all essential factors influencing the light interception and yield in cotton.

### 2.2. Experimental Design

Six cotton varieties were used in the treatments: CCRI60, SCRC28, Ji228, CCRI3799, OShi, and CCRI50. CCRI50 and OShi exhibited early maturity and a compact plant structure. Cultivars, such as CCRI60 and SCRC28, on the other hand, have smaller leaves, and a shorter growth period. The structure of CCRI3799 is relatively loose, with larger leaves and longer fruit branches. CCRI60, SCRC28, Ji228, and CCRI3799 were tower-shaped cultivars, while OShi and CCRI50 were tube-shaped cultivars. A RCBD was used to arrange the replicates of each cotton genotype. The plots were established at random, with cotton grown in 10 rows with a row spacing of 0.8 m that was consistent across all genotypes. Each cotton genotype was replicated three times, and each plot was 64 m^2^ in size, with length and width being 8 m. The soil was covered with plastic mulch to prevent the evaporation of soil moisture, and the seeds were displayed by hand. After some days of emergence, small holes were made in the plastic mulch, which was then removed. A base dose of 225 was administered to all plots.

### 2.3. Data Collection

#### 2.3.1. PAR Interception and Transmission in the Canopy

Light interception (LI) data were collected from 10.00 to 10.30 am every 15 days during the growing season at 30, 45, 60, 75, 90, 105, and 120 days of emergence (DAE) on days when there were no clouds. The spatial-grid sampling method was used to measure the transmitted Photosynthetically Active Radiation (tPAR) and the reflected radiation in multiple layers of the canopy with the help of a movable 1.0 m-line light sensor (LI-191SA,LI-COR,Lincoln,NE,USA) and a datalogger (LI-1400, LI-COR). The TPAR and RPAR were sampled starting from a row on the west side to a neighboring east row, and in five sections in each plot with horizontal distances of 0 cm, 20 cm, 40 cm, 60 cm, and 80 cm. Similarly, the whole cotton canopy was separated into consecutive strata from the lowermost strata then every 20 cm to the uppermost strata of the canopy, with several strata fluctuating in plant height. The following equations from Bai [10] were used to calculate a significant portion of the transmitted Photosynthetically Active Radiation (tPAR), the reflected portion of radiation, and the intercepted amount of Photosynthetically Active Radiation (iPAR):

(1) t PAR=ΤΡARΙΡAR 

(2) r ΡAR=RΡARΙΡAR

(3) i ΡAR=I PAR−TPAR−RPARIPAR=1−t PAR−rPAR

The effect of rPAR is ignored in this study. Equation (3) is simplified as follows:

(4) iPAR=1−tPAR

#### 2.3.2. Estimation of PAR Distribution in the Canopy

Several positions inside the canopy, the iPAR, and the tPAR values were measured using the subsequent calculation adapted from Li [21]:

(5) $$Ζ\left({Χ}_{0}\right)=\sum_{i=0}^{n}\lambda_{i}Ζ\left({Χ}_{i}\right)$$

where Z(X_0_) is the identified amount of PAR, $\lambda_{i}$ is the coefficient of the sample, and the neutral state = 1 was used.

Furthermore, the formula generated by Kriging is specified based on the minimum variance as follows:

(6) $$\sum_{i=0}^{n}\lambda_{i}r\left(x_{i,\;}\;x_{j}\right)+\phi =r\left(x_{i,}x_{0}\right)$$

where, according to Lagrangian, r (x_i_, x_0_) is the surveyed and quantified radiation captured, and the evaluated standard of X_0_ is the predicted fact as calculated with the help of the neutral value.

#### 2.3.3. Calculation of Accumulated tPAR within the Whole Canopy

The cumulative interception rate and the accrued tPAR throughout the canopies of each cotton variety used in this experiment was determined using the equation collected from Simpson 3/8 rules foundation based on the Surfer software (Golden Software Inc., Golden, CO, USA) [22]. The formula is as follows:

(7) $$A_{i}=\left(\;\frac{3\Delta x}{8}\left(G_{i,1}\right)+3G_{i,2}+3G_{i,3}+2G_{i,4}+...+2G_{i,\mathrm{ncol}-1}+{\;G}_{i,\mathrm{ncol}}\;\right)$$

(8) $$V\;\approx \;\frac{3\Delta y}{8}\;\left({\;A}_{1}+3A_{2}+3A_{3}+2A_{4}+...+2{\;A}_{\mathrm{ncol}-1}+{\;A}_{\mathrm{ncol}}\;\right)$$

In the above equation, (5, 3, 3, 2, 3, 3, 2, 1) is the coefficient vector, the vertical distance of grids is indicated by Δx, and the horizontal distance is specified as Δy; G (I,j) is the specified node number, and the specific cross-sectional area volume is the accumulated volume of light.

#### 2.3.4. Leaf Area Index (LAI), Biomass Accumulation and Duration of Growth Period

For this experiment, three cotton plants were collected from each plot for each replication, with the roots, stems, green leaves, and reproductive structures all being dissected. Following that, the leaf area was calculated for each plot with the help of a computerized scanner (Phantom p800xl, MiCROTEK) and an Image-Pro plus 7.0. The dissected samples were then placed in a 105 °C electronic fan supported oven for 30 min. Finally, the plant samples were desiccated at 80 °C for exactly 48 h to determine the dry matter content for each sample. During the whole cotton-growing period the cotton growth was briefly plotted every week to record the growth stages. The cotton growth was measured from the planting of the seed till until half of total amount of bolls had opened (Table 1).

### 2.4. Yield and Yield Component Determination

The seed cotton was collected three times from each plot by hand. Three times the lint yield was calculated and was subsequently ginned. Harvesting the open bolls at random was used to determine the yield components, such as boll weight and the percentage of lint. To estimate the boll weight the entire cotton seed of 100 bolls was divided by total no. of bolls. The percentage of lint was determined using the following formula:

$$\mathrm{Lint}\;\mathrm{percentage}=\frac{\mathrm{Lint}\;\mathrm{weight}}{\mathrm{Seed}\;\mathrm{cotton}\;\mathrm{yield}}$$

### 2.5. Statistical Analysis

The data used in this whole experiment were analyzed and the figures were created using Origin.2018 (Electronic Arts Inc, Redwood City, CA, USA). For the batch data processing, Stata 14 (StataCorp LP, College Station, TX, USA) was used. Surfer 18 was used to create contour maps displaying the cultivars’ light interception and competition ability (Golden Software Inc., USA).

## 3. Results

### 3.1. The Terrestrial Distribution Features of Canopy PAR

The iPAR values in the different locations throughout the canopy were evaluated using Surfer software V11, and the contour plots were fitted for the captured radiation. These plots presented the features of the spatial dissemination of the radiation in the canopy in a straightforward manner (Table 2).

**Figure 2:**
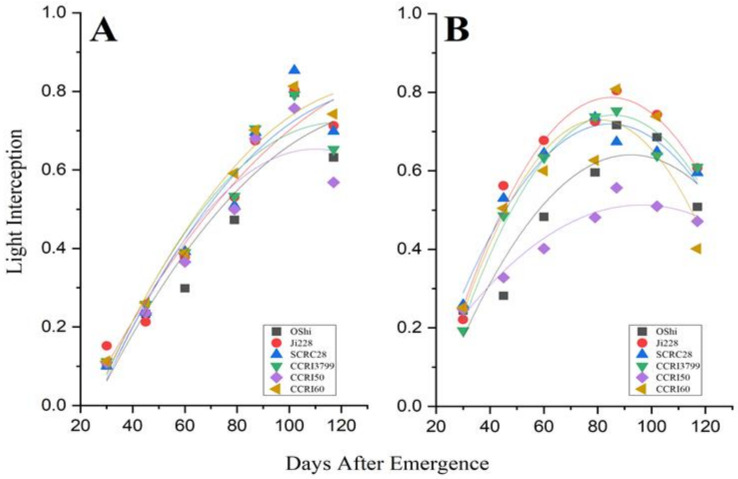
The distribution of Intercepted Photosynthetically Active Radiation (iPAR) during entire cotton-growing season for OShi, Ji228, SCRC28, CCRI3799, CCRI50, and CCRI60 in 2018 (**A**) and 2019 (**B**).

**Figure 3:**
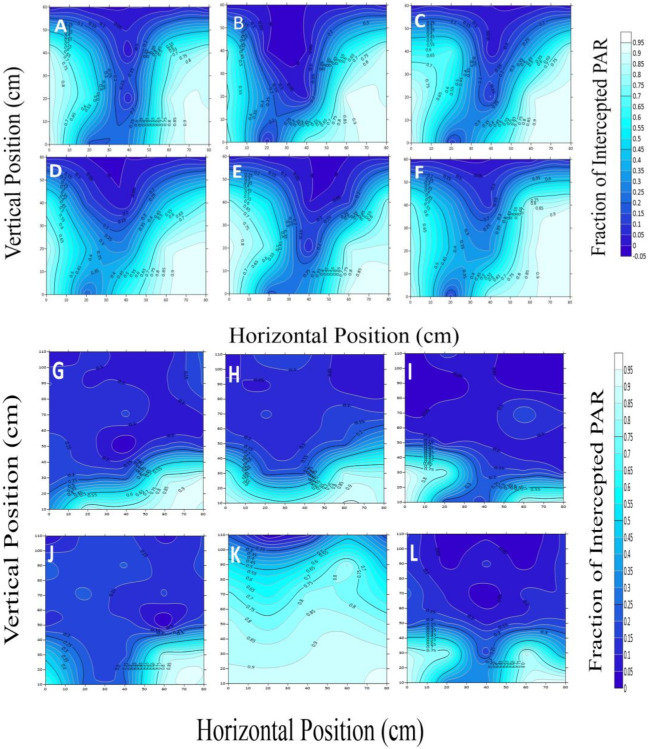
Vertical and horizontal distribution of iPAR at the squaring stage for OShi, Ji228, SCRC28, CCRI 3799, CCRI50, and CCRI60 in 2018 (**A**–**F**) and 2019 (**G**–**L**).

**Figure 4:**
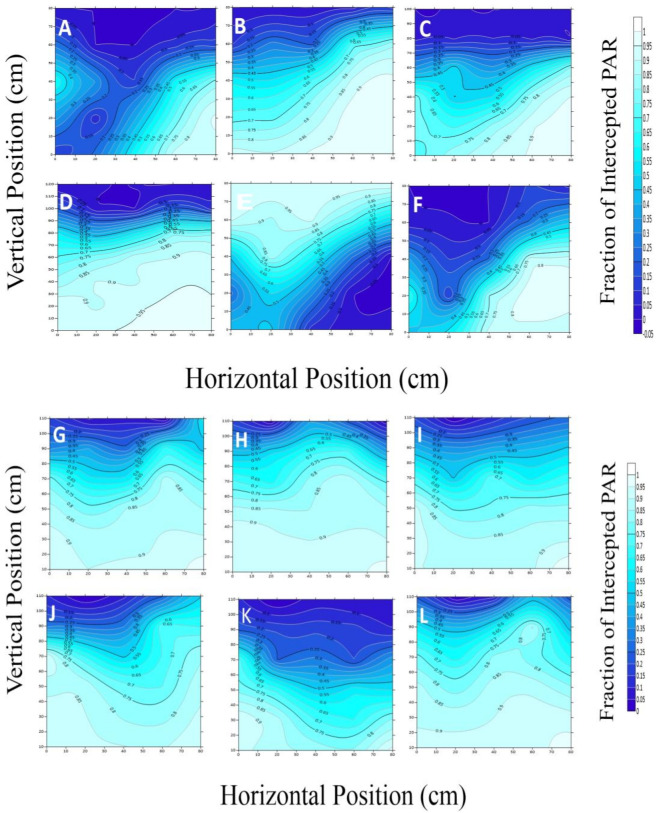
Vertical and horizontal distribution of iPAR at the blossoming and boll-forming stage for OShi, Ji228, SCRC28, CCRI 3799, CCRI50, and CCRI60 in 2018 (**A**–**F**) and 2019 (**G**–**L**).

The six cotton cultivars, OShi, Ji228, SCRC28, CCRI3799, CCRI50, and CCRI60 were used for a detailed presentation of the Intercepted Photosynthetically Active Radiation (iPAR) (Figure 2). We looked at the outcomes during two phases of cotton development: squaring, when the plants in the inner row are not yet covered, and blooming and boll-forming, when the canopy has already closed. Figures show the OShi, Ji228, CCRI3799, CCRI50, and CCRI60 spatial distributions of light interception (LI) in greater detail (Figure 3 and Figure 4).

The intercepted PAR of the cotton types used in this experiment and the cumulative days from sowing were fitted using a quadratic relationship, and the correlation coefficients were all greater than 0.72. Three distinct different cultivars, Ji228, CCRI50, and CCRI60 were chosen for describing the data in detail as representative cultivars. The light interception exhibited a downward opening quadratic parabola, and, furthermore, the values of “a” were negative in both years. On the other hand the correlation in 2019 was better than in 2018.

All the contour plots in this study behaved in a “V” shape during the early growing stage. For example, in 2018, the projected rate of iPAR fluctuated from 0.06–0.27 in the 20 cm vertical state and 0.57–0.91 in the 60 cm horizontal state, and in the adjacent rows of cotton when it was 0.02–0.76 in the 40 cm vertical state. However, the highest amount of iPAR was found at the 20 cm depth where the range of interception was 0.65–0.99. The values of CCRI60 were 0.11–0.43 and 0.84–0.88 at 20 cm and 60 cm in the vertical direction, respectively, whereas they were 0.07–0.80 at 40 cm in the horizontal direction. The maximum value appeared near the 20 cm direction with a range of 0.68–1.0. At 20 cm, 60 cm, and 40 cm of the CCRI50 canopies, the values were 0.17–0.67, 0.09–0.86, and 0.05–0.30, respectively. Similarly, with a value of 0.73–1.0, the highest PAR intermitted via a canopy closure depth of 20 cm.

The cotton branches started expanding simultaneously with the leaf area, resulting in the canopy being compact, which caused drastic changes in the structural dissemination of the iPAR. In the 20 cm and 60 cm horizontal positions, closest to the lines of cotton, the range of iPAR values, starting from the lowest point to the upper point of the canopy, were 0.09–0.93 (Ji228), 0.01–0.82 (CCRI60), and 0.01–0.94 (CCRI50). In the middle position (40 cm), Ji228, CCRI60, and CCRI50 were approximately 0.50–0.91, 0.09–0.77, and 0.09–0.94, respectively. In the opposite direction, the iPAR values in the Ji228 canopies were 0.01–0.85 and 0.02–0.93 at 20 cm and 60 cm, respectively. At the 40 cm vertical point, the middle lines, the iPAR values ranged from 0.18 to 0.91. At the bottom of the canopy of CCRI50 and CCRI60, the light interception ranged from 0.09–0.93 and 0.01–0.82, respectively. However, it was approximately 0.01–0.86 (CCRI50) and 0.20–0.76 at the mid-point (40 cm vertical position) (CCRI60).

### 3.2. LAI Development and Biomass Accumulation in Various Cultivars

**Figure 5:**
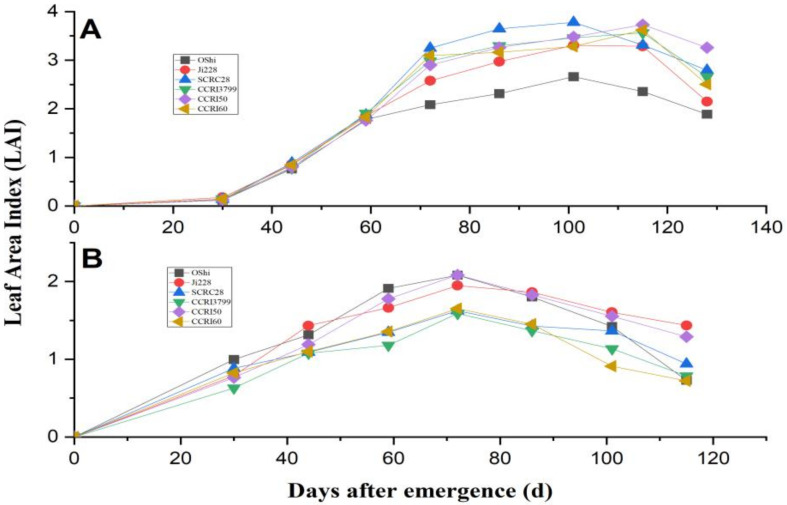
Leaf area index of six varieties in 2018 (**A**) and 2019 (**B**).

As a result of the diverse types of cotton cultivation, the leaf area index changes amid the varieties (Figure 5).

During the developmental period of cotton, LAI exhibited a quadratic trend with effective correlation coefficients in a range of 0.91–0.98 (Table 3).

The LAI increased with plant growth in the early developmental stage, then gradually decreased after reaching a peak due to lower leaf senescence. Using Ji 228, CCRI50, and CCRI60 as examples, the highest LAI values in 2018 were 1.94, 1.58, and 2.08, respectively, whereas they were 3.73, 2.65, and 3.77 in 2019. However, the peak times for LAI arrival were 72 days after sowing in 2018, and 115,101 and 101 in 2019.

The crop growth model accurately defined the evolution of the final dried mass, with significant coefficients ranging from 0.96-0.99 (Table 4). In the model presented here, the value of K indicates the maximum amount of dry mass that could be produced by the crops over the entire growing period. In 2018, CCRI60 obtained the highest (12,613.49) K value, whereas OShi had the lowest (8,442.43) value. Ji228 obtained the highest K value in 2019 and CCRI50, the compact cultivar, obtained the lowest K value. In 2018, SCRC28 produced 22.7%, 1.8%, 9.4%, 14.7%, and 0.9% higher total biomass than OShi, Ji228, CCRI3799, CCRI60, and CCRI50, whereas in 2019 Ji228 produced 41.5%, 25.4%, 22%, 41.2%, and 13.2%.

### 3.3. The Relationships and Fitted Models for Leaf Area Index, Total Dry Matter and Fraction of Intercepted PAR

**Figure 6:**
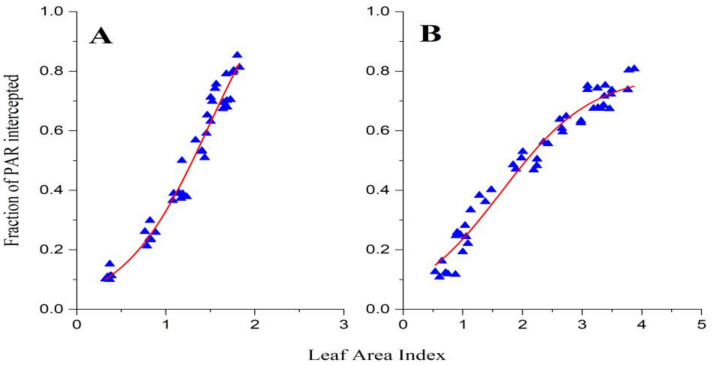
Relationship between LAI and iPAR in 2018 (**A**) and 2019 (**B**).

A logistic model adequately describes the relationship between light interception and the leaf area index as shown in (Figure 6).

For the entire growth period of the different cotton cultivars, LAI development exhibited a sigmoidal growth curve with a highly significant correlation coefficient of 0.96. The fitted equations for the intercepted PAR and the leaf area index are given below:

$$\mathrm{Equation}\;A\;2018\text{:}\;Y=1.231\text{⁄}(1+e^{-2.079\left(x-1.482\right)})\;\;\;\;\;R^{2}=0.96$$

$$\mathrm{Equation}\;B\;2019\text{:}\;Y=0.790\text{⁄}(1+e^{-1.315\left(x-1.646\right)})\;\;\;\;\;R^{2}=0.96$$

In the above equation, X represents LAI, and Y represents iPAR.

**Figure 7:**
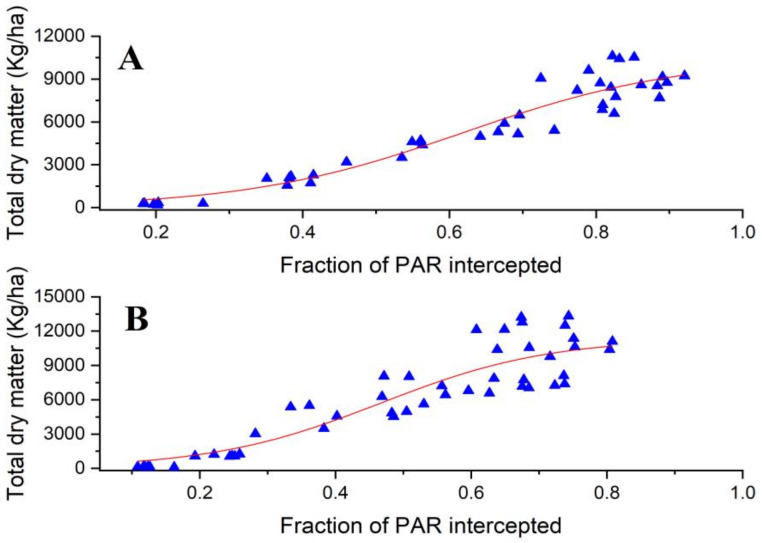
Relationship between the fraction of intercepted PAR and total dry matter accumulation during the whole growing period for six cultivars in 2018 (**A**) and 2019 (**B**).

There was also a sigmoidal curve, which describe the total dry matter accumulation, which obtained a significant correlation with light interception with R2 values of 0.91 and 0.82 in 2018 and 2019, respectively. To estimate the RUE, a logistic model was used to describe the links between the accumulated dry mass and the intercepted PAR (Figure 7). The fitted equations are given below:

$$\mathrm{Equation}\;C\;2018\text{:}\;Y=10496.23\text{⁄}(1+e^{-6.713\left(x-0.618\right)})\;\;\;R^{2}=0.91$$

$$\mathrm{Equation}\;D\;2019\text{:}\;Y=(11383.13)/(1+e^{-8.001\left(x-0.465\right)})\;\;R^{2}=0.82$$

In each equation, X represents the total dry matter and Y represents the iPAR.

### 3.4. Yield

In this study, the cotton yields ranged from 2506.1 to 3889.1 and 3086.3 to 4474.6 in 2018 and 2019, respectively. The cotton yield is significantly affected by the cotton genotypes. SCRC28 had the maximum seed cotton yields of 3889.1 and 4474.6 Kg ha^−1^ in 2018 and 2019, which were 25%, 16%, 20%, 36%, and 5% higher than OShi, Ji228, CCRI3799, CCRI50, and CCRI60 in 2018 and 15%, 3%, 15%, 31%, and 4% higher than OShi, Ji228, CCRI3799, CCRI50, and CCRI60 in 2019. In 2018, the maximum lint yields obtained were 1325.4 and 1627.1 Kg ha-1 for CCRI60 and Ji228, respectively, which were 10%, 8%, 3%, 19%, and 22% higher than those for OShi, Ji228, SCRC28, CCRI3799, and CCRI50, respectively. Furthermore, they are 7%, 4%, 7%, 23%, and 2% higher than those produced for OShi, SCRC28, CCRI3799, CCRI50, and CCRI60 in 2019. CCRI50 had the highest lint percentage (42.6%), followed by SCRC28, Ji228, CCRI3799, OShi, and CCRI60 (Table 5).

## 4. Discussion

The capability of the plant canopy to capture light is critical in determining plant biomass production and cotton growth. The light interception in diverse arrangements of canopies is difficult to identify and is biased by canopy architecture [23]. In different crops the arrangement of the canopy is influenced by the plant type [24]. Because of the higher LI during the boll-setting stage, the loose canopy structure captured a higher LI than cultivars containing a compact arrangement of the canopy in this study. The peak LI occurred later in 2019 owing to lower temperatures and low rainfall, which resulted in slower cotton growing and expansion. Loose-type cotton varieties had higher peaks of LAI in this study, which is the basic feature of LI of cotton [25]. The horizontal and vertical distribution of LI within the canopy varied significantly among the different varieties and canopy features. During the very early stages of development, the radiation transmission rate reduced with the penetration from the upside to the lower portion. Moreover, it reduced rapidly near rows than in between rows, lending to a credence in Bai’s findings (2016). The delayed stages of cotton growth in fully developed canopies showed statistically different spatial distributions of light with diverse plant patterns.

In both years, cotton with loose canopy structures captured a higher amount of light than congested varieties, demonstrating that the variation of LI in the entire canopy of diverse type cultivars was primarily due to the LI of the mid, upper, and outer canopy. Cotton varieties with loose structured canopies and longer branches generally achieved more LAI, which resulted in a lower canopy light transmittance, being less than that at the top- and mid-level canopy. The canopy’s LI is a key factor in evaluating the dry mass yield and growth of the cotton [26,27]. As a result, several prior studies concentrated on the canopy micro-environment and light improvement for increasing the inherent productivity of the crop [28,29]. The structure of a crop canopy can have a significant impact on its specific functions, for example stress tolerance and yield, besides which, the size of the canopy also has a crucial impact on the canopy architecture [30]. The effects of the crop canopy structure on radiation dissemination and interception beneath the canopy, in particular, are frequently examined [31].

In our experiment, cotton vegetative and reproductive biomass showed a high linear relationship with cumulative LI, which was consistent with the previous research [32,33,34]. The iPAR manifolded as an oval curve over the whole developmental time of the cotton in this study, where it was enhanced speedily through the vegetative growth and the leaf area index extension at a primary state, then decreased due to leafage senescence. These findings are consistent with those of Li and Xue [32]. In addition, we found a significant correlation between light interception and LAI, which is consistent with the invention of L.A. Vargas, G.A. Maddonni, and R.A. Ruiz [34,35,36]. However, the trends in both 2018 and 2019 were found to be almost same, representing that loose structured canopies obtained a higher LAI and biomass than compact structured varieties. The cotton yield significantly depended on the variety and canopy structure [37]. The cotton cultivars CCRI60, Ji228, and SCRC28 produced the highest cotton yields in both 2018 and 2019, indicating that cotton cultivars perform differently. Cotton production was discovered to be a combination of boll weight and the total boll number of cotton, along with the cultivar, which influences the comparative impacts of each [38]. Comparing the development of cotton cultivars over the two years revealed that environmental factors influenced the final photosynthetic products. Insufficient rainfall in 2018 hampered vegetative body growth and the gathering of photosynthetic yields. Excessive rainfall and a short period of sunshine during the reproductive growth resulted in leaf fall and low LAI values. Finally, cotton plants with a compact structure canopy with extended developmental periods intercepted a higher quantity of radiation and produced more biomass. As a result, cotton yield and yield distribution are extensively improved by suitable canopy structures.

## 5. Conclusions

The influence of cotton genotype and the plant canopy structure on light interception, yield components, and the link between biomass accumulation and light interception were investigated in this study. Optimizing the canopy structure and light is critical throughout the developmental season, and it is mandatory to investigate the local dissemination of the light interception for various plant types. According to the findings of this study: (i) cotton varieties with compact-structure canopies and tube-shape structures captured comparatively higher radiation than loose structured canopies and tower-shape cotton varieties, (ii) biomass accumulation is positively and linearly correlated with radiation interception, and (iii) LAI is highly and positively associated with cumulative PAR interception. Despite the fact that this study discussed the impacts of diverse cotton canopy structures on intercepted PAR, the photosynthetic production and attributes were not considered. Furthermore, because this experiment was carried out in a specific area, the Yellow River basin of China, the results are applicable in this region. Extensive researches and computer-based model evaluations are required to precisely describe the impact of cotton varieties on light interception and photosynthetic production.

## Figures and Tables

**Table 1:** Date of different growth stages of six cultivars in 2018 and 2019.

Year 2018					
	Seeding Date	Squaring Stage	Flowering Stage	Boll-Opening Stage	Duration (Days)
OShi	20-Apr	29-May	26-Jun	6-Aug	108
Ji228	20-Apr	31-May	26-Jun	11-Aug	113
SCRC28	20-Apr	29-May	25-Jun	7-Aug	109
CCRI3799	20-Apr	31-May	26-Jun	8-Aug	110
CCRI50	20-Apr	26-May	22-Jun	1-Aug	103
CCRI60	20-Apr	31-May	25-Jun	6-Aug	108
**Year 2019**					
OShi	18-Apr	1-Jun	27-Jun	16-Aug	120
Ji228	18-Apr	2-Jun	29-Jun	20-Aug	124
SCRC28	18-Apr	31-May	25-Jun	20-Aug	124
CCRI3799	18-Apr	31-May	29-Jun	19-Aug	123
CCRI50	18-Apr	22-May	15-Jun	7-Aug	111
CCRI60	18-Apr	2-Jun	27-Jun	22-Aug	126

**Table 2:** iPAR simulation equations for six cultivars in 2018 and 2019: Y = aX^2^ + bX + c.

	Year 2018, N = 7	R^2^	Year 2019, N = 7	R^2^
	Equation		Equation	
OShi	Y= −0.000057X^2^ + 0.01602X − 0.03672	0.88	Y= −0.00012X^2^ + 0.02215X − 0.38134	0.86
Ji228	Y= −0.000045X^2^ + 0.01443X − 0.29045	0.93	Y= −0.00017X^2^ + 0.03004X − 0.48937	0.96
SCRC28	Y= −0.000065X^2^ + 0.01777X − 0.40792	0.92	Y= −0.00014X^2^ + 0.02443X − 0.31470	0.95
CCRI3799	Y= −0.000081X^2^ + 0.01942X − 0.43106	0.93	Y= −0.00016X^2^ + 0.02909X − 0.50655	0.97
CCRI50	Y= −0.000091X^2^ + 0.02017X − 0.45775	0.89	Y= −0.00064X^2^ + 0.01244X − 0.08539	0.94
CCRI60	Y= −0.000067X^2^ + 0.01806X − 0.39894	0.96	Y= −0.00019X^2^ + 0.03127X − 0.52644	0.84

Y: Estimated value of iPAR; X: Days after sowing.

**Table 3:** LAI simulation equations for six cultivars in 2018 and 2019: Y = aX^2^ + bX + c.

	Year 2018	R^2^	Year 2019	R^2^
	Equation		Equation	
OShi	Y = −0.00032X^2^ + 0.04392X − 0.08444	0.92	Y = −0.00068X^2^ + 0.13385X − 3.48763	0.94
Ji228	Y = −0.00029X^2^ + 0.04712X − 0.10468	0.95	Y = −0.00054X^2^ + 0.12217X − 3.27755	0.97
SCRC28	Y = −0.00043X^2^ + 0.05881X − 0.1593	0.92	Y = −0.00070X^2^ + 0.14243 − 3.79326	0.96
CCRI3799	Y = −0.00026X^2^ + 0.0404X − 0.05105	0.96	Y = −0.00070X^2^ + 0.14147 − 3.7355	0.94
CCRI50	Y = −0.00026X^2^ + 0.03937X − 0.10683	0.91	Y = −0.00054X^2^ + 0.1063X − 2.68308	0.98
CCRI60	Y = −0.00031X^2^ + 0.04948X − 0.16102	0.91	Y = −0.00080X^2^ + 0.15809 − 4.21312	0.95

Y: Estimated value of LAI; X: Days after sowing.

**Table 4:** Biomass accumulation simulation equation for six cultivars in 2018 and 2019: Y = K/(1 + ae^bx^).

	Year 2018		Year 2019	
	Equation	R^2^	Equation	R^2^
OShi	Y = 8442.43475/(1 + 212.37688e^−0.07082X^)	0.99	Y = 11166.84/(1 + 147.69962e^−0.06458X^)	0.98
Ji228	Y = 10932.71558/(1 + 111.96196e^−0.06114X^)	0.98	Y = 24945.82/(1 + 135.04495e^−0.04143X^)	0.98
SCRC28	Y = 10887.38529/(1 + 115.46423e^−0.06098X^)	0.97	Y = 13404.76/(1 + 1485.80753e^−0.08578X^)	0.96
CCRI3799	Y = 9238.54557/(1 + 249.42951e^−0.07848X^)	0.98	Y = 14920.98/(1 + 126.1678e^−0.04995X^)	0.96
CCRI50	Y = 9593.09873/(1 + 127.34178e^−0.06266X^)	0.99	Y = 10283.46/(1 + 538.99072e^−0.07553X^)	0.97
CCRI60	Y = 12613.49407/(1 + 43.72154e^−0.04241X^)	0.97	y = 16753.47/(1 + 123.76809e^−0.04891X^)	0.97

Y: Estimated value of biomass accumulation; X: Days after sowing.

**Table 5:** Comparison of seed cotton and lint yields of various cotton varieties during the growing seasons in 2018 and 2019.

Treatment	Seed Cotton Yield	Lint Yield	Lint Percentage
**Year 2018**			
**OShi**	2929.8 ± 51.7e	1187.0 ± 43.4b	39.3 ± 0.47abc
**Ji228**	3282.3 ± 32.0c	1219.4 ± 43.4ab	40.2 ± 0.41ab
**SCRC28**	3889.1 ± 87.5a	1289.4 ± 94.1ab	38.3 ± 2.10c
**CCRI3799**	3115.9 ± 70.3d	1067.9 ± 61.2c	39.5 ± 0.73abc
**CCRI50**	2506.1 ± 75.9f	1037.8 ± 56.6c	40.9 ± 0.19a
**CCRI60**	3696.1 ± 19.0b	1325.4 ± 78.9a	38.7 ± 1.01bc
**Year 2019**			
**OShi**	3786.1 ± 18.1c	1513.4 ± 56.4b	41.4 ± 0.42ab
**Ji228**	4318.4 ± 57.2b	1627.1 ± 63.8a	42.1 ± 0.62ab
**SCRC28**	4474.6 ± 83.5a	1565.9 ± 80.6ab	42.1 ± 0.81ab
**CCRI3799**	3808.3 ± 54.1c	1513.9 ± 40.7b	41.7 ± 0.78ab
**CCRI50**	3086.3 ± 48.9d	1249.3 ± 78.7c	42.6 ± 0.25a
**CCRI60**	4295.1 ± 77.6b	1595.9 ± 53.6ab	40.9 ± 1.41b

Means followed by the same letters within the same category are statistically similar according to Duncan’s multiple range test at *p* < 0.05.

## Data Availability

Not applicable.

## References

[1] Chaudhry U.F., Khalid M.N., Aziz S., Amjad I., Khalid A., Noor H., Sajid H.B. (2022). Genetic studies in different F2 segregating population for yield and fiber quality traits in cotton (Gossypium hirsutum L.). Int. J. Agri. Biosci..

[2] Zafar M.M., Manan A., Razzaq A., Zulfqar M., Saeed A., Kashif M., Khan A.I., Sarfraz Z., Mo H., Iqbal M.S.J.A. (2021). Exploiting Agronomic and Biochemical Traits to Develop Heat Resilient Cotton Cultivars under Climate Change Scenarios. Agronomy.

[3] Zafar M.M., Zhang Y., Farooq M.A., Ali A., Firdous H., Haseeb M., Fiaz S., Shakeel A., Razzaq A., Ren M.J.A. (2022). Biochemical and Associated Agronomic Traits in Gossypium hirsutum L. under High Temperature Stress. Agronomy.

[4] Bashir M.H., Nawaz M.S., Khan A.Z., Aziz S., Ullah F., Qasim M. (2022). Characterization and advancement of microsatellite (SSR) markers for various stresses in wheat. Int. J. Agri. Biosci..

[5] Khan M.M., Shakeel Nawaz M., Saeed A., Khan M.A. (2022). Combining ability estimates of various morphological and quality traits of okra. Int. J. Agri. Biosci..

[6] Hassan A., Nasser A., Shahani A.A.A., Aziz S., Khalid M.N., Mushtaq N., Munir M.A. (2022). Assessment of fiber and yield related traits in mutant population of cotton. Int. J. Agri. Biosci..

[7] Zafar M.M., Jia X., Shakeel A., Sarfraz Z., Manan A., Imran A., Mo H., Ali A., Youlu Y., Razzaq A.J.F.i.P.S. (2021). Unraveling Heat Tolerance in Upland Cotton (*Gossypium hirsutum* L.) Using Univariate and Multivariate Analysis. Front. Plant Sci..

[8] Acreche M.M., Briceño-Félix G., Sánchez J.A.M., Slafer G. (2009). Radiation interception and use efficiency as affected by breeding in Mediterranean wheat. Field Crop. Res..

[9] Reta-Sánchez D.G., Fowler J.L. (2002). Canopy Light Environment and Yield of Narrow-Row Cotton as Affected by Canopy Architecture. Agron. J..

[10] Bai Z., Mao S., Han Y., Feng L., Wang G., Yang B., Zhi X., Fan Z., Lei Y., Du W. (2016). Study on Light Interception and Biomass Production of Different Cotton Cultivars. PLoS ONE.

[11] Jiang Y., Li C., Paterson A.H. (2016). High throughput phenotyping of cotton plant height using depth images under field conditions. Comput. Electron. Agric..

[12] Feng G., Luo H., Zhang Y., Gou L., Yao Y., Lin Y., Zhang W. (2016). Relationship between plant canopy characteristics and photosynthetic productivity in diverse cultivars of cotton (*Gossypium hirsutum* L.). Crop. J..

[13] Brodersen C.R., Vogelmann T.C. (2010). Do changes in light direction affect absorption profiles in leaves?. Funct. Plant Biol..

[14] Wünsche J.N., Lakso A.N., Robinson T.L., Lenz F., Denning S.S. (1996). The Bases of Productivity in Apple Production Systems: The Role of Light Interception by Different Shoot Types. J. Am. Soc. Hortic. Sci..

[15] Louarn G., Lecoeur J., Lebon E. (2007). A Three-dimensional Statistical Reconstruction Model of Grapevine (Vitis vinifera) Simulating Canopy Structure Variability within and between Cultivar/Training System Pairs. Ann. Bot..

[16] Monteith J.L. (1977). Climate and the efficiency of crop production in Britain. Philos Trans. R. Soc. Lond. B Biol. Sci..

[17] Vos J., Evers J., Buck-Sorlin G.H., Andrieu B., Chelle M., De Visser P.H.B. (2010). Functional–structural plant modelling: A new versatile tool in crop science. J. Exp. Bot..

[18] Rey H., Dauzat J., Chenu K., Barczi J.-F., Dosio G.A.A., Lecoeur J. (2008). Using a 3-D Virtual Sunflower to Simulate Light Capture at Organ, Plant and Plot Levels: Contribution of Organ Interception, Impact of Heliotropism and Analysis of Genotypic Differences. Ann. Bot..

[19] Sinoquet H., Thanisawanyangkura S., Mabrouk H., Kasemsap P. (1998). Characterization of the Light Environment in Canopies Using 3D Digitising and Image Processing. Ann. Bot..

[20] Melo J.D., Carreno E.M., Calviño A., Padilha-Feltrin A. (2014). Determining spatial resolution in spatial load forecasting using a grid-based model. Electr. Power Syst. Res..

[21] Li Y., Mao S., Feng L., Han Y., Wang G., Fan Z., Sun E. (2012). Spatial distribution characteristics of photosynthetic active radiation in cotton canopy based on geo-statistics. Trans. Chin. Soc. Agric. Eng..

[22] Zhi X., Han Y., Mao S., Wang G., Feng L., Yang B., Fan Z., Du W., Lü J., Li Y. (2014). Light Spatial Distribution in the Canopy and Crop Development in Cotton. PLoS ONE.

[23] Mariscal M., Orgaz F., Villalobos F. (2000). Modelling and measurement of radiation interception by olive canopies. Agric. For. Meteorol..

[24] Xing F., Han Y., Feng L., Zhi X., Wang G., Yang B., Fan Z., Lei Y., DU W., Wang Z. (2018). Genotypic variation in spatiotemporal distribution of canopy light interception in relation to yield formation in cotton. J. Cotton Res..

[25] Reynolds M.P., B M.D., Gutiérrez-Rodríguez M., Saavedra A.L. (2000). Photosynthesis of wheat in a warm, irrigated environment: I: Genetic diversity and crop productivity. Field Crop. Res..

[26] Chenu K., Franck N., Dauzat J., Barczi J.-F., Rey H., Lecoeur J. (2005). Integrated responses of rosette organogenesis, morphogenesis and architecture to reduced incident light in Arabidopsis thaliana results in higher efficiency of light interception. Funct. Plant Biol..

[27] Escobar-Gutiérrez A.J., Combes D., Rakocevic M., de Berranger C., Eprinchard-Ciesla A., Sinoquet H., Varlet-Grancher C. (2009). Functional relationships to estimate Morphogenetically Active Radiation (MAR) from PAR and solar broadband irradiance measurements: The case of a sorghum crop. Agric. For. Meteorol..

[28] Fila G., Sartorato I. (2011). Using Leaf Mass per Area as predictor of light interception and absorption in crop/weed monoculture or mixed stands. Agric. For. Meteorol..

[29] Gonias E.D., Oosterhuis D.M., Bibi A.C. (2011). Light interception and radiation use efficiency of okra and normal leaf cotton isolines. Environ. Exp. Bot..

[30] Jiang Y., Li C., Paterson A.H., Sun S., Xu R., Robertson J. (2018). Quantitative Analysis of Cotton Canopy Size in Field Condi-tions Using a Consumer-Grade RGB-D Camera. Front. Plant Sci..

[31] Watanabe T., Hanan J.S., Room P.M., Hasegawa T., Nakagawa H., Takahashi W. (2005). Rice Morphogenesis and Plant Architecture: Measurement, Specification and the Reconstruction of Structural Development by 3D Architectural Modelling. Ann. Bot..

[32] Xue H., Han Y., Li Y., Wang G., Feng L., Fan Z., Du W., Yang B., Cao C., Mao S. (2015). Spatial distribution of light interception by different plant population densities and its relationship with yield. Field Crop. Res..

[33] Zarate-Valdez J.L., Whiting M.L., Lampinen B.D., Metcalf S., Ustin S., Brown P. (2012). Prediction of leaf area index in almonds by vegetation indexes. Comput. Electron. Agric..

[34] Vargas L., Andersen M., Jensen C., Jørgensen U. (2002). Estimation of leaf area index, light interception and biomass accumulation of Miscanthus sinensis ‘Goliath’ from radiation measurements. Biomass Bioenergy.

[35] Maddonni G., Otegui M. (1996). Leaf area, light interception, and crop development in maize. Field Crop. Res..

[36] Ruiz R., Bertero H. (2008). Light interception and radiation use efficiency in temperate quinoa (Chenopodium quinoa Willd) cultivars. Eur. J. Agron..

[37] Girma K., Teal R.K., Freeman K.W., Boman R.K., Raun W.R. (2007). Cotton lint yield and quality as affected by applications of N, P, and K fertilizers. J. Cotton Sci..

[38] Wang Y., Ye G., Luan N., Xiao J., Chen Y., Chen D. (2009). Boll size affects the insecticidal protein content in Bacillus thuringiensis (Bt) cotton. Field Crop. Res..

